# Intrapartum Acupuncture Provision in the UK: A Survey of Independent Acupuncture Practitioners

**DOI:** 10.3390/healthcare14152321

**Published:** 2026-08-01

**Authors:** David J. Carr

**Affiliations:** 1 Division of Maternal-Fetal Medicine, Department of Obstetrics, Gynecology and Reproductive Sciences, UVM Larner College of Medicine, University of Vermont, Burlington, VT 05405, USA; d.j.carr@alumni.ucl.ac.uk; 2 Department of Maternal and Fetal Medicine, UCL Institute for Women’s Health, University College London, London WC1E 6AU, UK

**Keywords:** acupuncture, labor pain management, survey, intrapartum care, integrative obstetrics, integrative maternity care, obstetric acupuncture

## Abstract

**Background/Objectives:** Acupuncture is effective for labor pain yet rarely used in the UK. Midwife-delivered acupuncture (commonplace in Europe) is cost-effective but typically demands a protocol-based approach. Randomized controlled trial (RCT) protocols have varied greatly in terms of acupuncture rationale/style, needle number/location and retention time. The aim of this study was to determine the intrapartum acupuncture experience of professional UK acupuncturists specializing in pregnancy/childbirth and compare their treatment recommendations with research evidence regarding acupuncture “dose” during labor. **Methods:** Fifty self-identified obstetric acupuncturists (various styles) with 10 [4, 6] years of experience (median [interquartile range]) were surveyed about their personal intrapartum acupuncture experience and recommendations regarding traditional acupuncture point selection and overall treatment approach. **Results:** Of 50 respondents, 36% (n = 18) had never attended a labor. For those with experience, the median number of labors attended was 0.4/year [interquartile range 0.1–0.3]. Only 12% (n = 6) had attended ≥10 labors. Recommendations included using between 2 [1, 2] and 8 ± 4 (mean ± standard deviation) needles left in situ for 5 [1, 10] to 30 [30, 30] min. Only 32% (n = 16) advised retention throughout labor, of which 25% (n = 4) would tape needles down. The remainder advised interval treatments, typically 1–2 hourly. Most practitioners highlighted the need to vary the treatment according to individual needs/sensitivity. Regarding points, only SP6 and LI4 were ubiquitous, although 41/51 RCT points were mentioned, which were most often segmental and/or so-called major points for central effects (SP6/LI4/BL32/BL60/LR3/BL67/PC6/BL31/ST36/Shenmen). Regarding needle stimulation, 18% (n = 9) recommended none, 20% (n = 10) elicitation of de qi only, and 54% (n = 27) repetitive manual manipulation (every 5–15 min). Only 8% endorsed electroacupuncture use. **Conclusions**: UK experience with intrapartum acupuncture appears limited. Most practitioners recommended short periods of needling using relatively few needles, i.e., low-dose acupuncture.

## 1. Introduction

Acupuncture—a treatment modality involving the insertion of filiform needles into the body—is becoming increasingly accepted as a method of pain relief in Western nations. In the UK, acupuncture is currently recommended by the National Institute for Health and Care Excellence (NICE) for the management of migraine, tension-type headache and chronic pain [1,2], for which it is available on the National Health Service (NHS). In the USA, it is recommended by the American College of Physicians for the treatment of low back pain [3], for which it is now reimbursed by the Center for Medicare & Medicaid Services [4]. There is also a growing body of evidence in support of the safety and effectiveness of acupuncture during pregnancy [5,6,7,8,9], including in labor and delivery [10,11,12]. The most recent Cochrane review and meta-analysis, which included 13 randomized controlled trials (RCTs) of intrapartum acupuncture, found a significant reduction in pain intensity compared with usual care, with a standardized mean difference (Cohen’s d value) of −1.31 (95% confidence interval (CI) −0.49 to −2.14), indicating a medium-to-large effect size. Acupuncture also decreased the use of pharmacologic analgesia relative to usual care (risk ratio 0.72; 95% CI 0.60–0.85). Clinical heterogeneity among the included studies—for example, with respect to the use of manual acupuncture versus electroacupuncture (EA)—plus an observation that elicitation of “de qi” (a characteristic needling sensation experienced secondary to needle manipulation in the tissues) was associated with increased patient satisfaction scores [13], raises important questions about acupuncture “dose” requirements for labor pain management. Acupuncture dose reflects the degree of stimulation of the nervous system during treatment and is determined by: (1) the number of needles used; (2) the retention time; and (3) the degree of needle stimulation, either manual or electrical [14]. As summarized in Table 1, these factors varied significantly across 21 identified RCTs of penetrating acupuncture in labor, with insertion of anywhere between two [15,16,17,18,19,20,21] and twenty-one needles [22], retention for just a few minutes [23] up to the whole duration of labor [13,15,23,24]—sometimes with needles being taped down [15,23,24,25]—and stimulation ranging from a single elicitation of de qi sensation [13,15,23,24,25] to strong EA [18,20,26,27]. Regarding traditional acupuncture point selection (i.e., anatomical location), RCT protocols have consistently incorporated needling segmental to the uterus (T11–L2 or S2–S4 based on its sympathetic or parasympathetic innervation, respectively) ventrally (e.g., CV3, CV4), distally (e.g., SP6, KI3, LR3) or dorsally (e.g., BL23, BL28, BL31–34) ± so-called “major” point locations (e.g., LI4, ST36, PC6), stimulation at which is believed to induce strong central effects.

Most of the largest RCTs to date have been conducted in European countries, including Denmark, Norway and Sweden, where acupuncture is routinely provided during labor in many maternity units [35]. In these settings, treatment is typically delivered by midwives, and this is likely to be cost-effective given that they are already providing one-to-one care to parturients, so there are no additional staffing costs. By contrast, in the USA, it has been reported that acupuncture is only rarely used during childbirth, usually by private arrangement between a laboring patient and licensed acupuncturist (LAc), and almost always in the context of birth outside of a hospital [36]. Similarly, in the UK, obstetric acupuncture is generally provided by private practitioners rather than on the NHS. However, a pilot integrated obstetric acupuncture service has been established within a central London maternity unit, where a large number of midwives have been trained to needle clients during labor using a protocolized acupuncture approach [37]. In developing evidence-based guidelines to support this novel NHS service, a survey of UK intrapartum acupuncture providers was conducted to supplement the available RCT data (as detailed in Table 1). The primary aim of this survey was to determine the level of intrapartum experience of professional UK acupuncturists specializing in pregnancy/childbirth. The secondary aims were to explore the degree to which this group’s professional experience and perceptions of best practices reflect the protocols of the various RCTs conducted to date and the dose of acupuncture that practitioners might administer in labor.

## 2. Materials and Methods

### 2.1. Survey Population

Invitations to an online survey (using the Survey Monkey platform) were sent in 2015 to professional acupuncturists self-identifying as specialists in pregnancy and childbirth via membership of a UK-wide continuing professional development group known as the Acupuncture Childbirth Team (ACT). None of these individuals were midwives. Contact was made via email circulation lists and ACT Facebook pages for the London and Hertfordshire groups, and practitioners were provided with a link to follow to complete the survey if interested. Of note, no individual emails were sent, so a precise response rate could not be calculated. However, response rate was estimated to be >80% based on ACT London/Herts group numbers at the time of the survey. In accordance with national guidance and in keeping with the literature on research governance for surveys [38], written informed consent and institutional ethical approval were not sought for this survey, as it included no identifiable information fields and was completely voluntary. Prior to completing the online survey, participants were made aware of the aims of the research and of the associated risks, which were considered to be negligible given that no sensitive or personal information was collected. At the time the survey was completed, ACT membership required a formal qualification in the practice of acupuncture, followed by a minimum of 14 h of structured postgraduate training specifically in obstetrics, including recognition of “red flags” to support safe practice.

### 2.2. Survey Content

Acupuncturists were asked about their general acupuncture experience (current years in practice and personal style/rationale, e.g., Traditional Chinese medicine (TCM), Western medical acupuncture (WMA), Five Element, etc.) and experience of intrapartum acupuncture (reflected by the number of labors attended in the capacity of an acupuncture treatment provider). Respondents were also asked specific questions about their preferred treatment approach with a specific emphasis on the determinants of acupuncture dose, including the number of needles used (minimum and maximum), retention time (minimum and maximum) and degree of stimulation (none, one time elicitation of de qi, repeated manual stimulation (MS) or EA). Those that reported using MS were additionally asked how often they would manipulate the needles, and those favoring the use of EA were asked whether they used high (80–100 Hz), low (2–10 Hz) or alternating frequencies. Practitioners were also presented with a list of the 51 different acupuncture points used in published RCTs of intrapartum acupuncture (Table 1) and asked to select those that they had personally incorporated into their clinical practice and/or would recommend for laboring patients. Respondents were also encouraged to make additional point recommendations (outside of the prescribed list of 51 points) and any general or specific comments in a free text box (with no restrictions). Finally, each practitioner was asked to select their “top five” traditional acupuncture point preferences for each of the following five domains, representing general and specific considerations for treatment during labor/delivery: (1) analgesia; (2) relaxation; (3) augmentation of uterine contractions; (4) fetal malposition (e.g., persistent occipito-posterior (OP) or transverse (OT) position; and (5) nausea ± vomiting.

### 2.3. Statistical Analysis

Normality of distribution was assessed using the Shapiro–Wilk test. Data are presented as mean ± standard deviation (SD) if normally distributed and median [interquartile range] if skewed. Descriptive statistical analysis was carried out using the Statistical Package for the Social Sciences (SPSS) version 21.0 (SPSS Inc., Chicago, IL, USA).

## 3. Results

### 3.1. Intrapartum Acupuncture Experience

**Figure 1:**
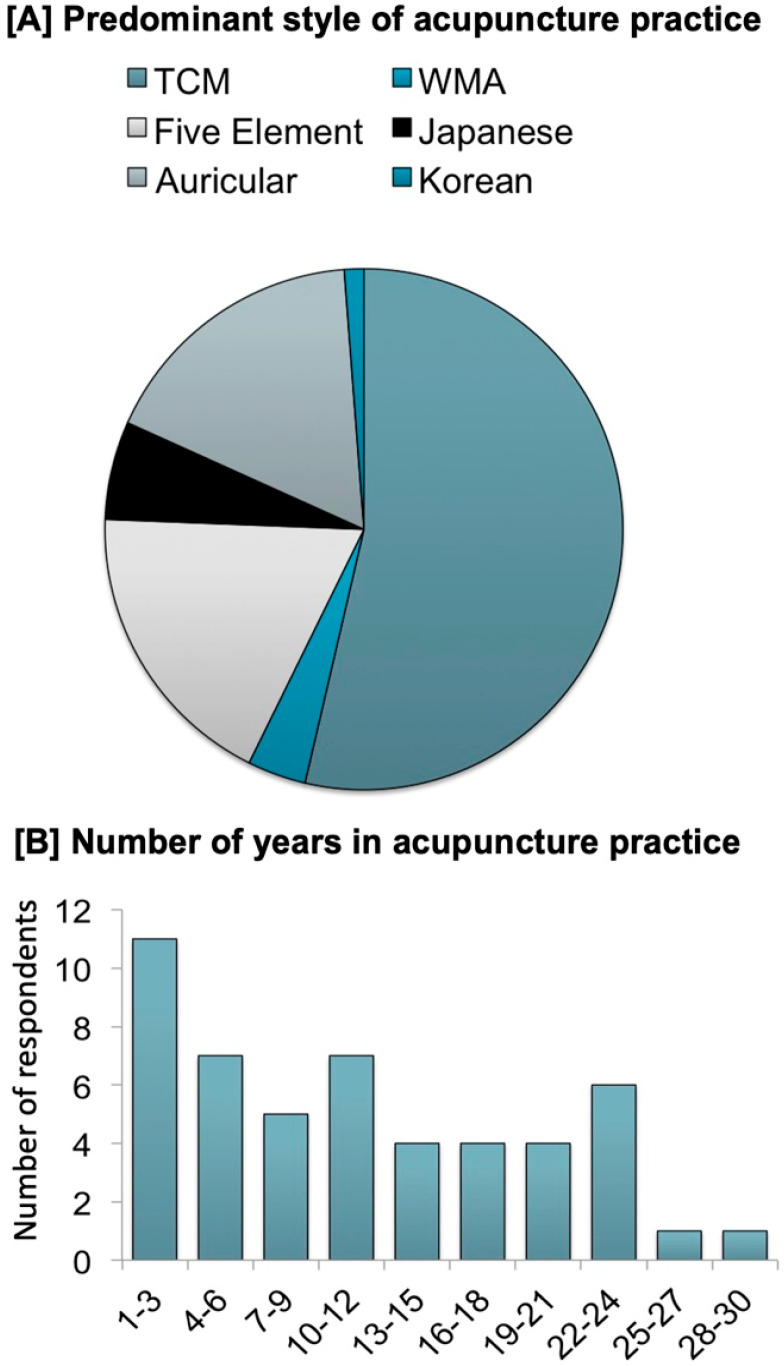
Acupuncture styles [pie chart, (**A**)] and number of years in practice [histogram, (**B**)] of 50 self-identified obstetric acupuncturists completing an online survey about their intrapartum experience. Abbreviations: TCM = traditional Chinese medicine; WMA = Western medical acupuncture. Of note, practitioners could choose more than one style—pie chart represents distribution of n = 82 selections.

**Figure 2:**
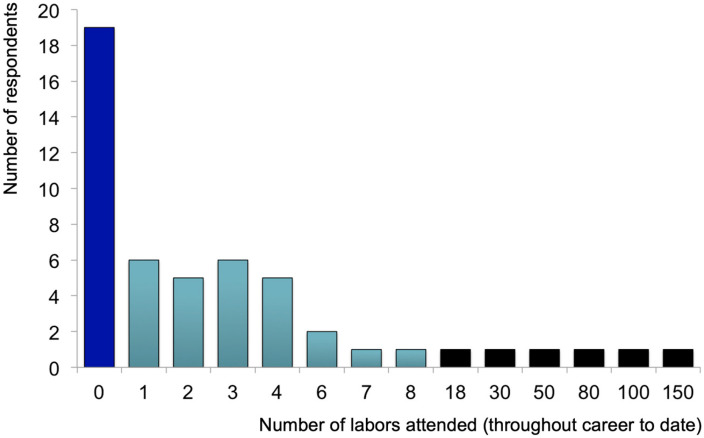
Bar chart demonstrating number of labors attended (during their career to date) by 50 self-identified obstetric acupuncturists completing an online survey about their intrapartum experience. The blue bar indicates those with no prior intrapartum acupuncture experience. The teal and black bars represent those having attend <10 and ≥10 labors, respectively.

A total of 50 practitioners completed the survey. There were no incomplete responses and no apparent duplicate submissions. As shown in Figure 1A, the practitioners surveyed had been practicing for 10 [4, 6] years (range 1 to 30 years). As shown in Figure 1B, individual acupuncture styles included TCM (88%, n = 44), Five Element (30%, n = 15), auricular (28%, n = 14), Japanese (10%, n = 5), WMA (6%, n = 3) and Korean hand acupuncture (2%, n = 1). Overall, 36% (n = 18) of respondents had never attended a labor as an acupuncture provider (Figure 2). Amongst those who had previously provided intrapartum care (n = 32), the average number of labors attended per year (estimated by dividing the number of labors attended to date by the number of years in practice) was 0.4 [0.1–0.3]. Only 12% (n = 6) had attended ≥10 labors during their career to date.

### 3.2. Determinants of Acupuncture Dose

**Figure 3:**
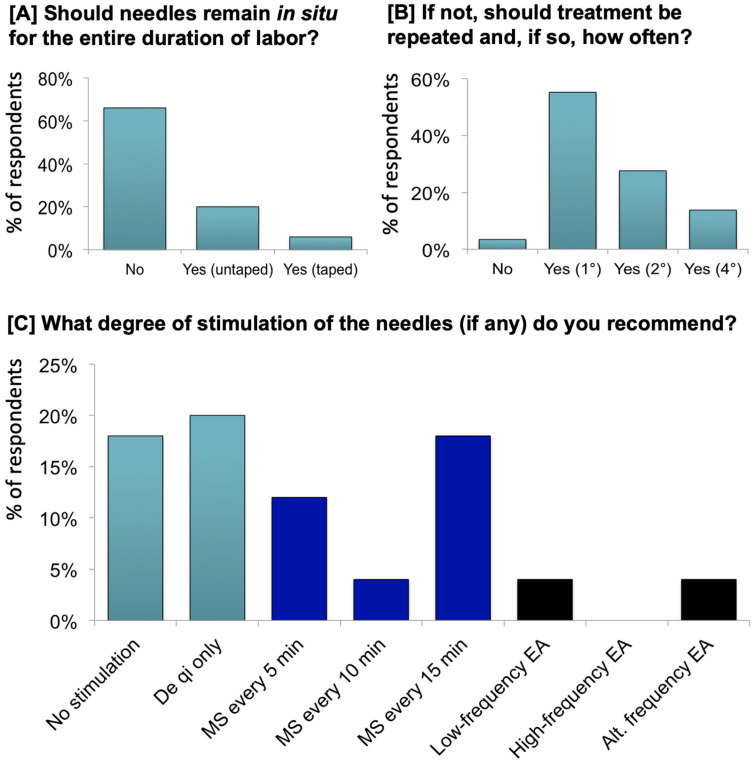
Treatment recommendations regarding the retention of needles throughout labor (**A**), frequency of repeat acupuncture treatments (when needles not left in situ) (**B**) and type and degree of needle stimulation (**C**) from 50 self-identified obstetric acupuncturists completing an online survey about their intrapartum experience. Abbreviations: MS = manual stimulation; EA = electroacupuncture; ° = hourly. In panel (**C**), the blue and black bars indicate variations of MS and EA, respectively.

Regarding the determinants of acupuncture dose, practitioners recommended insertion of a minimum of 2 [1, 2] needles (range 1 to 5) and maximum of 8 ± 4 needles (range 2 to 16). As illustrated in Figure 3A, only 32% of respondents (n = 16) advised keeping the needles in situ for the full duration of labor, of which only 25% (n = 4) felt that they should be taped down. The remaining 68% of respondents (n = 34) advised that the needles should be left in situ for a minimum of 5 [1, 10] min (range 1 to 20 min) and maximum of 30 [30, 30] min (range 1 to 45 min), and all but one advised that the treatment should be repeated, typically at 1–2 hourly intervals (Figure 3B). Regarding degree of needle stimulation, 18% (n = 9) recommended none, 20% (n = 10) the elicitation of de qi once only and 54% (n = 27) some degree of MS, respectively (Figure 3). It was recommended that MS should be repeated at intervals of 5 to 15 min (Figure 3C). Only four respondents (8%) endorsed EA, all of whom recommended low-frequency stimulation either alone or in combination with high-frequency stimulation in an alternating fashion. No respondents recommended continuous high-frequency EA in isolation. In the free text comments, it was notable that many acupuncturists stated that they would tend to vary the treatment approach according to the individual needs and sensitivity of each patient.

### 3.3. Traditional Acupuncture Point Recommendations

**Figure 4:**
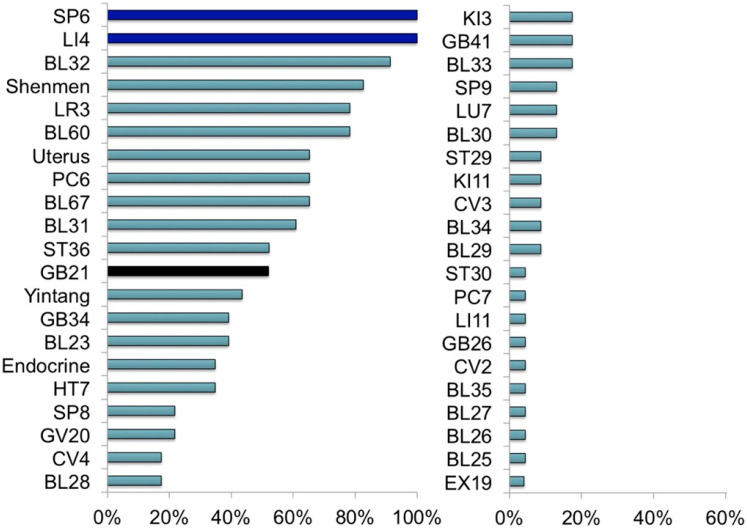
Traditional acupuncture point utilization/recommendations from 50 self-identified obstetric acupuncturists completing an online survey about their intrapartum experience. Respondents were asked to select from a list of 51 traditional acupuncture point locations that had previously been used in randomized controlled trials of acupuncture for labor and were allowed to nominate extra points by free text entry (indicated by black bars). Ubiquitous responses are indicated by blue bars. Shenmen refers to the auricular point.

Regarding traditional acupuncture point locations, only SP6 and LI4 were ubiquitous across all respondents; however, 41 of the 51 points used in the RCTs had reportedly been needled in practice on one or more occasions by this particular group of practitioners (Figure 4). The ten most commonly utilized traditional acupuncture point locations were SP6 (100%, n = 50), LI4 (100%, n = 50), BL32 (92%, n = 46), BL60, LR3 and ear Shenmen (all 80%, n = 40), BL67 and PC6 (64%, n = 32), BL31 (58%, n = 29) and ST36 (54%, n = 27). Only one additional point was nominated via free text, namely GB21, which was mentioned by 50% of respondents (n = 25) despite not having formed part of the points protocol of any RCT to date. The five most popular acupuncture point locations (based on the individual rankings) for specific indications are detailed in Table 2. These mainly included different combinations of the top ten general points, as detailed above, with the additional nomination of Yintang, GV20 and HT7 for relaxation ± nausea and vomiting, and GB34 and SP8 for fetal malposition, respectively.

## 4. Discussion

Firstly, the findings of the present study suggest that intrapartum acupuncture provision by UK independent practitioners is lacking. Of this sample of 50 acupuncturists specializing in pregnancy and childbirth, approximately one third had never attended a labor in order to provide acupuncture treatment. The remaining two thirds of respondents reported treating, on average, only one laboring woman every two years (and a maximum of seven per year). This is in stark contrast to rates of uptake in European countries, such as Sweden and Germany, where intrapartum acupuncture is routinely provided in up to 94–97% of obstetric units [35,39,40] and is reportedly used in up to 25% of all labors [41]. Given the obvious logistic challenges facing the establishment of robust individual arrangements for intrapartum acupuncture provision in the private sector, it is perhaps unsurprising that it seldom appears to work in practice. The inherent uncertainty around the timing of onset and duration of labor effectively requires the practitioner to ensure 24 h on call availability around the time of the estimated date of delivery. Practitioners are also highly likely to need to work during the night and/or cancel scheduled daytime clinics at very short notice, which may have significant financial implications and cause considerable inconvenience to other clients. Furthermore, the potential cost to the client of prolonged attendance by a private practitioner in labor may be prohibitive. Some of these issues could arguably be overcome by employing a resident acupuncturist. However, although this was piloted relatively successfully in a New York City hospital [42], it has not been widely adopted to date. An arguably more feasible model involves the training of midwives to integrate acupuncture within their scope of practice, which is commonplace across Europe and is not associated with extra staffing costs. In Germany, obstetric acupuncture is predominantly delivered by midwives [39,40] and is included in the undergraduate curriculum of 43% of midwifery schools nationwide [43]. In Sweden, around 75% of midwives have received acupuncture training, which is frequently funded by their employers [35,41]. As a general rule, European midwives are trained in WMA techniques through specific intrapartum courses that may last anywhere from one day to 10 weeks [23,37,44].

With respect to acupuncture treatment recommendations, the surveyed practitioners endorsed 80% of the traditional acupuncture point locations that have been incorporated into RCT protocols. Although the rationale they provided was generally classical (due to the predominance of TCM and other traditional styles among those surveyed), there was strong agreement with European RCT protocols [15,22,23,25,28,30], which generally involve individualized acupuncture point selections based on neurophysiological principles. By contrast, 90% of RCTs conducted in China have used fixed-point protocols with no individualization of treatment [16,17,18,19,20,21,26,33,34]. Of these, six used only SP6 ± LI4. This suggests that a protocol-driven approach is acceptable for intrapartum care, even amongst proponents of TCM. In this survey, only SP6 and LI4 were selected by all respondents. Notably, SP6 has been used in every single one of the 21 intrapartum RCTs to date and has oftentimes been the only location stimulated (albeit always using EA) in the Chinese studies [16,17,18,20,21]. Stimulation of somatic afferent nerves within flexor digitorum longus and/or the posterior tibial nerve by needling at SP6 can result in sacral neuromodulation (at the S2 level) in the form of afferent competition at the dorsal horn and somato-autonomic effects that may modulate uterine pain and contractility, respectively [45,46]. EA at SP6 alone during labor is associated with reductions in both pain scores and the length of the first stage of labor [18,19,21]. Stimulation at LI4 is classically associated with strong analgesic effects, which may be mediated via the central nervous system (CNS). EA at LI4 modulates specific brain regions associated with pain processing [47] and has been demonstrated to have a beneficial effect on labor progress when used as an adjunct to intravenous oxytocin for delay in the first stage of labor [48]. It has been demonstrated in rats that EA at both LI4 and SP6 stimulates the dynorphin/κ-opioid system in the lumbar spinal cord [49], which may be responsible for some of its analgesic effects. However, other segmental, heterosegmental and central mechanisms may also be important, for example endogenous opiate release in the CNS, enhanced descending pain inhibition, and deactivation of the limbic system [50].

Regarding determinants of acupuncture dose, participant responses were intended to be hypothesis-generating only and so should be interpreted with caution. Nevertheless, respondents to this survey generally seemed to favor “low-dose” acupuncture. This is supported by their suggestions of a relatively small number of needle insertions (average 2–8) and relatively short retention times (average 5–30 min) with repeat treatments as necessary. This arguably mirrors the trials that have just used a few fixed points, usually SP6 only [16,17,18,19,20,21] or SP6 in combination with LI4 [26]. However, despite the widespread use of EA across these trials, most respondents advised MS (at intervals of 5–15 min). This may represent unfamiliarity or lack of experience with EA rather than reluctance to use it per se, although this was not assessed by the questionnaire administered herein. Interestingly, Vixner et al. compared high-frequency EA (80 Hz) versus manual acupuncture and MS (at intervals of 10 min) in a three-armed RCT and found that EA (but not manual acupuncture) significantly reduced rates of epidural anesthesia [22]. However, it has not been established whether EA is more effective, or indeed whether higher dose treatments (in general) have greater therapeutic efficacy. Ultimately, in the absence of appropriate training and availability of equipment to perform EA, regular MS would seem to be a reasonable compromise. Relatively few practitioners surveyed in this study would advise needle retention throughout the whole duration of labor, and only 8% in total would endorse taping needles down. This is consistent with temporal trends in RCT protocols, wherein only the early trials used needle taping [15,23,24,25]. By contrast, more recent RCTs have favored intermittent treatment, which promotes mobilization during labor. This may reflect the growing awareness that adopting an upright posture and ambulating in labor reduces the length of the first stage as well as rates of epidural anesthesia and Cesarean section [51].

There are a number of significant limitations to this study that need to be acknowledged. First, the sample size was very small, given the intent was to specifically target individuals most likely to have intrapartum experience in the UK setting (i.e., those with a self-declared interest/specialization in this area), which is known to be a small community of practice. It remains unknown how many acupuncturists not affiliated with ACT attend labors and whether their treatment recommendations might differ from those surveyed herein. Moreover, the predominance of TCM practitioners means the findings may not extrapolate well to other acupuncture styles commonly used in European intrapartum settings, where midwives typically utilize neurophysiologically based protocols. Accordingly, there is a risk of selection bias and restricted generalizability. Second, given the lack of intrapartum experience among the survey respondents, their treatment recommendations need to be interpreted with caution (especially as they presumably represent a mixture of experiential reporting for those who had previously provided treatment in labor versus aspirational reporting for those who had not). Third, there is a risk of recall bias, as well as potential response bias. In particular, given that the survey response rate could not be precisely calculated, the degree of potential non-response bias is unknown. The fact that the practitioners chose whether or not to access the survey also introduces self-selection bias and, although each of the 50 survey responses were confirmed to be unique, the possibility of duplicate submissions cannot be fully ruled out. Fourth, the questionnaire was not validated or pilot tested prior to administration. Notably, it did not include details of other procedural aspects of acupuncture that further influence dose, such as laterality of needling, duration of MS, or depth of needling (which is likely to differ by point location). Fifth, no clinical outcome data was collected (as there was no way to verify the accuracy of any clinical claims by the responding practitioners). Sixth, the survey lacked a priori registration and a pre-specified statistical analysis plan (although no inferential statistical analysis was performed and the findings are presented in descriptive terms only). Finally, the study design does not allow any conclusions to be drawn about optimal treatment approaches, as agreement between practitioner opinion and clinical trial protocols does not ultimately validate the clinical effectiveness of intrapartum acupuncture.

## 5. Conclusions

In summary, clinical experience with intrapartum acupuncture among UK practitioners appears limited. Protocol-based acupuncture provision by trained midwives may increase access to this potentially beneficial therapy in maternity units. Despite some emerging evidence that EA may be superior to manual acupuncture (with or without manual stimulation), most practitioners surveyed herein recommended intermittent, brief periods of needling using a relatively small numbers of needles, i.e., “low-dose” acupuncture. However, whether this truly represents best practice for intrapartum acupuncture remains unknown, especially given the relative lack of real-world clinical experience among this group of practitioners.

## Figures and Tables

**Table 1:** Determinants of acupuncture dose in intrapartum acupuncture trials (n = 3820 participants).

Study (First Author, Year)	Sample Size	Country	Treatment Provider	No. of Needles	Needle Retention Time	Degree of Needle Stimulation
Skilnand, 2002 * [15]	210	Norway	Midwife	2 to 21	20 min or until delivery (taped)	De qi only
Ramnerö, 2002 * [25]	100	Sweden	Midwife	NR	1–3 h (taped)	De qi only
Nesheim, 2003 * [23]	198	Norway	Midwife	NR	Few min or until delivery (taped)	De qi only
Ziaei, 2006 [24]	90	Iran	LAc	12	Until delivery (taped)	De qi only
Zhang, 2006 # [16]	120	China	LAc	2	30 min	EA
Zhou, 2007 # [17]	111	China	LAc	2	30 min	EA
Fan, 2007 * [26]	36	China	LAc	4	20 min	EA (2/100 Hz, 14–30 mA)
Hantoushzadeh, 2007 * [13]	150	Iran	LAc	NR	Until delivery (taped)	De qi only
Mårtensson, 2008 * [28]	128	Sweden	Midwife	9 to 12	40 min	De qi + MS q 10 min
Tjung, 2008 # * [27]	50	Philippines	LAc	4	20 min	EA (2/100 Hz, 14–30 mA)
Huang, 2008 * [29]	324	China	LAc	NR	30 to 60 min	EA (2/100 Hz)
Borup, 2009 * [30]	607	Denmark	Midwife	NR	30 to 120 min	NR (but no EA)
Ma, 2010 [18]	349	China	LAc	2	30 min	EA (2/100 Hz, 20 mA)
Ma, 2011 * [19]	350	China	LAc	2	30 min	EA (4/20 Hz)
Mackenzie, 2011 * [31]	105	UK	LAc	8	30 to 60 min	Intermittent MS or EA (2 Hz)
Liu, 2012 [21]	111	China	LAc	2	20 min	EA
Vixner, 2014 * [22]	303	Sweden	Midwife	13 to 21	40 min	De qi + MS q 10 min, or EA
Dong, 2015 * [20]	188	China	LAc	2	30 min	EA (2/100 Hz, 5–40 mA)
Asadi, 2015 [32]	63	Iran	LAc	4	20 min	De qi + MS q 5 min
Xiao, 2019 [33]	127	China	LAc	8	25 min	EA (2/50 Hz, 0.1–1.0 mA)
Cheng, 2025 [34]	100	China	NA	6	NA	NA

* Included in most recent Cochrane review [12]. # Information extracted from systematic reviews [10,11]. Abbreviations: EA = electroacupuncture; q = every; LAc = licensed acupuncturist; MS = manual stimulation; NA = not available; NR = not reported. Trials of non-penetrating interventions (e.g., acupressure, auriculotherapy using seeds/studs, transcutaneous electrical nerve stimulation (TENS), transcutaneous electrical acupuncture point stimulation (TEAS), moxibustion) and administration of substances or materials (e.g., pharmacopuncture, bee venom acupuncture, catgut embedding) at traditional acupuncture point locations were excluded. Only RCTs of penetrating acupuncture are included.

**Table 2:** Practitioners’ traditional acupuncture point recommendations (ranked) for specific indications in labor.

Ranking	Analgesia	Relaxation	Augmentation	Malposition	Nausea/ Vomiting
1st	LI4	Yintang	SP6	BL67	PC6
2nd	LR3	Ear Shenmen	LI4	BL60	ST36
3rd	BL32	HT7	BL32	SP6	LR3
4th	SP6	GV20	BL31	GB34	GV20
5th	BL31	PC6	LR3	SP8	HT7

## Data Availability

The raw data supporting the conclusions of this article will be made available by the author on request.

## Abbreviations

The following abbreviations are used in this manuscript:

ACT	Acupuncture Childbirth Team
EA	Electroacupuncture
CNS	Central nervous system
IQR	Interquartile range
LAc	Licensed acupuncturist
MS	Manual stimulation
NHS	National Health Service
NICE	National Institute for Health and Care Excellence
NA	Not available
NR	Not reported
OP	Occipito-transverse
OT	Occipito-posterior
RCT	Randomized controlled trial
SD	Standard deviation
SPSS	Statistical Package for the Social Sciences
TCM	Traditional Chinese medicine
UK	United Kingdom
USA	United States of America
WMA	Western medical acupuncture
